# Project 'BANGED’ – A Bedside Tool to Aid Post Head Banging Reviews

**DOI:** 10.1192/bjo.2025.10354

**Published:** 2025-06-20

**Authors:** Jemima Cohen, Sathya Vishwanath

**Affiliations:** Humber Teaching NHS Foundation Trust, Hull, United Kingdom

## Abstract

**Aims:** Project ‘BANGED’, a Quality Improvement Project (QIP) aimed to enhance confidence, consistency, and clarity, when completing post headbanging reviews (PHBR).

The world of psychiatry is often the first-time (and perhaps only time) resident doctors (RDs) are exposed to such behaviour thus request. This can be daunting, often inducing a ‘CT head reflex reaction’.

A tool to strike balance between true neurology vs over medicalisation seemed pressing. Thus, the bedside tool ‘BANGED’ was created. A guiding acronym for RDs to use, designed for inpatient settings. Aimed at the general adult population, however, has relevance to other areas such as Intellectual Disability.

QIP carried out at Humber Teaching NHS Foundation Trust (HTNFT).

‘BANGED’

Each letter represents key areas of focus for PHBRs and is as follows:

B – bruising, bumps (swelling), breakage of skin, bleeding (? active).

A – awareness – any LOC, GCS, awareness of triggers – reason for head banging if known (any ways of reducing this).

N – Neurological deficits – any red flags for head injury & Nausea/vomiting, are neurological observations required? Nursing engagements.

G – gross (motor) movements, gait.

E – eyes (pupils) equal and reactive to light, accommodation, any diplopia.

D – dizziness, drowsiness – don’t forget glucose (if dizzy and oral intake concerns).

**Methods:** 2024 timeline.

August: Created the acronym ‘BANGED’ following brief narrative review, discussion amongst psychiatry trainees and own experience. Showcased tool via integration of ‘BANGED’ into poster and presentation.

September: Gathered baseline data via pre-intervention questionnaire – sent out to all HTNFT psychiatry RDs – initial confidence, understanding, applicability of tool. Presented tool in teaching session. Distributed poster and displayed in staff facing areas on HTNFT inpatient units.

November: Shared results of pre-intervention questionnaire. Re-shared tool. Post Intervention questionnaire – gathered feedback regarding tool implementation into practice.

**Results:** Pre-Intervention Questionnaire:

Delivered face to face.

31 doctors responded of mixed grades.

Around half had never completed a PHBR (coincided with beginning of rotation).

19.4% selected ‘Not confident at all’ with such task.

93.5% were unaware of any helpful tools.

100% answered yes to ‘Would a tool such as an acronym help your approach?’.

Post-Intervention Questionnaire:

Delivered online.

9 doctors responded of mixed grades.

Most used the tool.

100% would recommend.

Comments: easy to use, relevant to clinical practice, clever acronym, improved confidence.

**Conclusion:** PHBRs remain a daunting yet apparent task for psychiatry RDs. The bedside tool ‘BANGED’ shows promise for improving approach, by offering guidance for key areas of focus.

Future practice – further cycles required, delivered in person – better response rate.

